# Meta-Analysis on the Association of ALDH2 Polymorphisms and Type 2 Diabetic Mellitus, Diabetic Retinopathy

**DOI:** 10.3390/ijerph14020165

**Published:** 2017-02-08

**Authors:** Guang-Yi Li, Zi-Bo Li, Fang Li, Li-Ping Dong, Liang Tang, Ju Xiang, Jian-Ming Li, Mei-Hua Bao

**Affiliations:** 1 Department of Anatomy, Histology and Embryology, Institute of Neuroscience, Changsha Medical University, Changsha 410219, China; liguangyi1977@163.com (G.-Y.L.); li-evans@hotmail.com (F.L.); ddongliping@163.com (L.-P.D.); tlcool318@163.com (L.T.); xiang.ju@foxmail.com (J.X.); 2 Department of Medical Laboratory, Changsha Medical University, Changsha 410219, China; lzb3039@126.com

**Keywords:** ALDH2 rs671, type 2 diabetic mellitus, diabetic retinopathy, polymorphism, meta-analysis

## Abstract

Type 2 diabetic mellitus (T2DM) is a disease with high prevalence and a major cause for death worldwide. Diabetic retinopathy (DR) is one of the major manifestation of diabetes. Aldehyde dehydrogenease 2 (ALDH2) detoxifies aldehyde produced during ethanol metabolism and oxidative stress. It has been found that the polymorphism in ALDH2 rs671 is probably associated with the risk of T2DM and DR. However, a lot of inconsistency and controversy still exists. In order to get a more precise and comprehensive estimation for the association between ALDH2 polymorphism with the risk of T2DM and DR, we conducted the present meta-analysis. A comprehensive literature search was conducted using databases, such as Pubmed, Embase, Cochrane Central Register of Controlled Trials, Chinese National Knowledge Infrastructure, and Chinese Biomedical Literature Database, for all related studies. The included studies met the inclusion criteria, such as being case-control studies about the association of ALDH2 polymorphism and T2DM or DR susceptibility, with sufficient data for the present analysis. Eight studies with 2374 cases and 6694 controls were involved in the present meta-analysis. The results indicated a significant lower risk of T2DM for *1/*1 genotype in homozygous models (*1/*1 vs. *2/*2, OR = 0.31, 95% CI = 0.11–0.89, *p* = 0.03) and in the dominant model (*1/*1 vs. *2/*2 + *1/*2, OR = 0.61, 95% CI = 0.37–1.00, *p* = 0.05). Subgroup analysis by ethnicity found a significant lower risk of T2DM in Chinese in all genotype models. No significant relation was found between ALDH2 rs671 and DR. In conclusion, the current meta-analysis indicated that ALDH2 rs671 was significantly related with T2DM. The ALDH2 rs671 might be able to be used as a predictor for the risk of T2DM. However, due to the existence of heterogeneity and publication bias in the involved studies, our results should be interpreted with caution.

## 1. Introduction

Type 2 Diabetic mellitus (T2DM) is a major risk factor for many diseases such as coronary artery disease (CAD) and ocular disorders [1,2]. During the pathogenesis of T2DM, many risk factors and genetic variants are involved, such as drinking, hyperlipidemia, etc. [3]. Among these factors, the associations between alcohol drinking and T2DM have been widely studied [4,5]. More alcohol consumption may relate to higher susceptibility to T2DM. Interestingly, East Asians are more likely to have alcoholism than Caucasians. This difference between ethnicity is partly due to the polymorphisms of acetaldehyde dehydrogenase 2 (ALDH2).

ALDH2 is a key enzyme for alcohol metabolism. It is encoded by the ALDH2 gene in chromosome 12. There is a significant single-nucleotide polymorphism (SNP) in ALDH2, named rs671 (G-to-A), which causes a replacement of glutamate to lysine at position 504. The Glu504 is named *1, and the 504Lys is named *2. The ALDH2 *2 allele causes a drastic decrease of enzyme activity [6]. ALDH2 is an enzyme which detoxifies reactive aldehydes, such as methylglyoxal and 4-hydroxynonenal. These aldehydes usually come from lipids and glucose [7,8,9]. It is reported that methylglyoxal and 4-hydroxynonenal can cause protein carbonylation and mitochondrial dysfunction, forming advanced glycation end products (AGEs) [10,11]. Therefore, the defect of ALDH2 caused by allele *2 may result in the accumulation of aldehydes and be related with the higher risk of T2DM. Many studies have described retinopathy as a main manifestation of diabetes. Furthermore, studies also found that diabetic retinopathy development is associated with the ALDH2 polymorphism [12,13].

Up to the present, several studies have demonstrated the relationship between ALDH2 rs671, T2DM, and diabetic retinopathy (DR). However, the results are controversial. Some of them reported a lower susceptibility of T2DM for the *1 allele, while the associations between them were not significant in other studies. To get a more precise and comprehensive estimation for the association between ALDH2 polymorphism with the risk of T2DM and DR, we conducted the present meta-analysis.

## 2. Methods

### 2.1. Literature Search and Inclusion Criteria

All the literature in the electronic databases Pubmed, Embase, Cochrane Central Register of Controlled Trials (CENTRAL), Chinese National Knowledge Infrastructure (CNKI), and Chinese Biomedical Literature Database were checked systematically and comprehensively. The search terms used were as follows: (Diabetes, or Diabetic Mellitus, or Diabetic retinopathy), (ALDH2, or aldehyde dehydrogenase 2), and (polymorphism, or SNP, or single nucleotide polymorphism). The literature in English or Chinese were included, and the search deadline for publications was 24 September 2016. All publications from the databases which met the search criteria were screened carefully, and the references and citations of included studies were checked for other potentially relevant studies. The PubMed option ‘Related Articles’ was also checked for additional studies.

The inclusion criteria were as follows: (1) case-control study; (2) study was about the association of ALDH2 polymorphism and diabetic mellitus or diabetic retinopathy susceptibility; (3) the data in the studies were sufficient for the present analysis. The exclusion criteria were as follows: (1) repeat publications, abstracts, dissertations, or reviews; (2) studies violating any of the inclusion criteria.

### 2.2. Data Extraction

The information from each included study was extracted by two investigators manually. The extracted information includes: first author’s name, publishing year, country, ethnicity, genotype method, the source of control, and case or control numbers of each genotype. All discrepancies that happened during the data extraction process were resolved by a consensus achieved by a third author.

### 2.3. Quality Evaluation

The quality of each involved study was evaluated according to previous studies [13,14,15]. Briefly, the representativeness of cases, representativeness of controls, ascertainment of diseases or control, genotyping examination, Hardy-Weinberg Equilibrium (HWE), association assessment, and total sample size were taken into account and given a corresponding score. Total scores ranged from 0 to 15. Two authors evaluated the quality of each study independently. If any discrepancy was found, a consensus was achieved by a third author.

### 2.4. Statistical Methods

The HWE of control group polymorphisms was evaluated by χ^2^-test, and the results with *p* < 0.05 were considered to be HWE deviations. We used the crude odds ratio (OR) with 95% confidence interval (CI) to assess the association between ALDH2 rs671 and T2DM risk, as well as DR risk. For the meta-analysis, pooled ORs were calculated in homozygous (*1/*1 vs. *2/*2), heterozygous (*1/*1 vs. *1/*2), dominant (*1/*1 vs. *1/*2 + *2/*2) and allelic models (*1 vs. *2), and the statistical significance was determined by the Z-test. If the results were *p* < 0.05, it was considered to be statistically significant. Subgroup analysis by sample size was conducted. Groups with total samples less than 1000 were treated as small and all other groups were treated as large.

The *I*^2^ test, which was considered to be independent of the number of studies in the meta-analysis, was used to evaluate the statistical heterogeneity between studies [16]. The heterogeneity among the studies was divided into high (*I*^2^ > 50%), moderate (25% > *I*^2^ > 50%), and low (*I*^2^ < 25%). If the studies had high or moderate heterogeneity, random-effects model (the DerSimonian and Laird method) was used; otherwise, the fixed-effect model (Mantel-Haenszel method) was used instead. To evaluate the stability of the results, and to assess the effect of individual study on pooled results, sensitivity analysis was performed. Begg’s funnel plot and Egger’s linear regression method were used to detect the publication bias. Results with *p* < 0.05 was considered to be statistically significant [17]. All statistical analyses were performed using the STATA 12.0 software (StataCorp, College Station, TX, USA) and Revman 5.3 (Cochrane Collaboration, London, UK).

## 3. Results

### 3.1. Literature Search and Characters of Involved Studies

**Figure 1:**
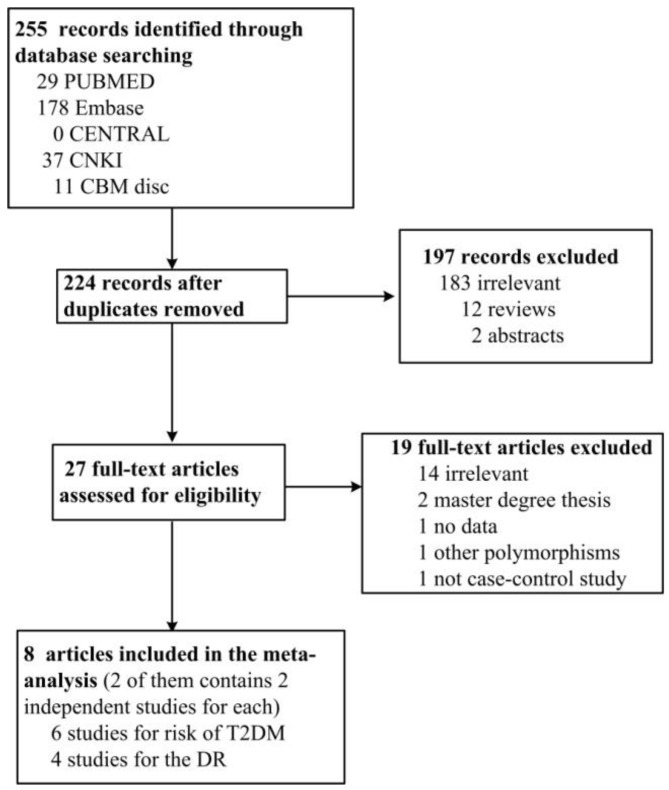
PRISMA flow chart for the inclusion and exclusion of searched studies.

In total, 224 articles were identified after duplicates were removed. Among them, 197 were found to be irrelevant, 12 were reviews, 2 were abstracts, 2 were Master’s degree dissertations, 1 did not have enough data, 1 studied a different polymorphism in ALDH2, and 1 was not a case-control study; after removal of these articles, 8 articles were included in the review. Among these 8 articles, the work from Maimaitikuerban [18] and Xu [19] used different controls (normal control and CAD patients without DM control) for the comparison between control and T2DM patients; therefore, they were considered as two independent studies. The studies using CAD patients without DM control were presented as Maimaitikuerban (1) and Xu (1) in our analyses. At last, 6 studies were about the associations between ALDH2 rs671 and T2DM, and 4 were about the association between ALDH2 rs671 and DR. The present meta-analysis included 2374 cases and 6694 controls in all. Table 1 and Table 2 show the characteristics of included studies, and Figure 1 is the PRISMA flow chart for the inclusion and exclusion of searched studies.

### 3.2. Results of Meta-Analysis

**Figure 2:**
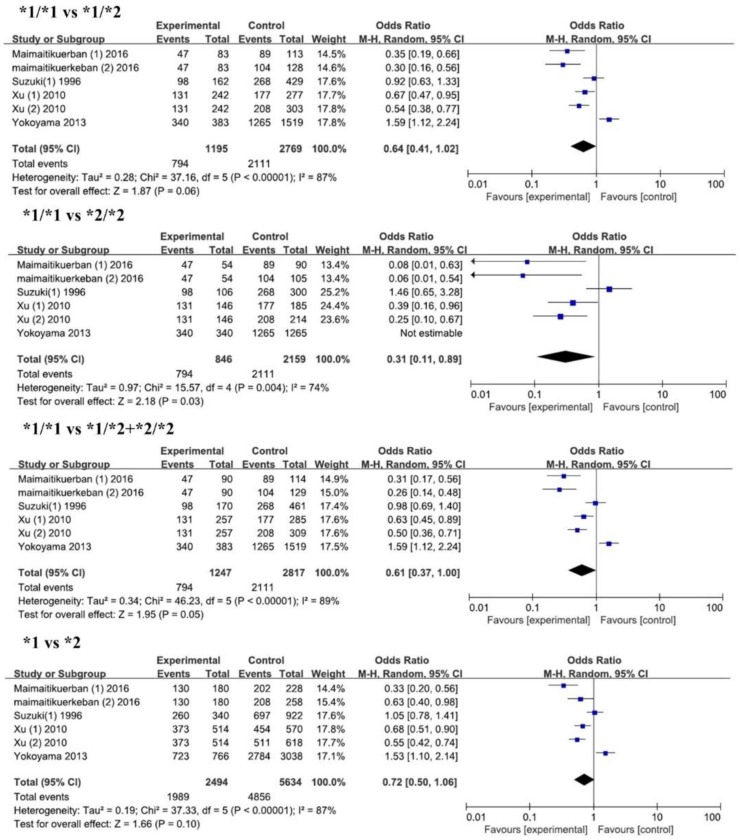
Forest plots of odds ratio for the association between ALDH2 rs671 and risks of T2DM.

**Figure 3:**
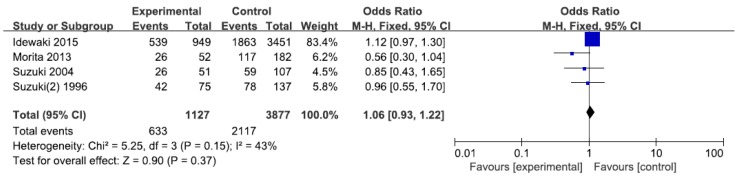
Forest plots of odds ratio for the association between ALDH2 rs671 and risks of DR.

As shown in Table 3 and Figure 2, we identified 6 studies with 1247 cases and 2817 controls for the association between ALDH2 rs671 and T2DM risks. In overall analysis, we found a significantly lower risk of T2DM in the *1/*1 genotype with an odds ratio of 0.31 compared with that of the *2/*2 genotype (95% CI = 0.11–0.89). When we conducted subgroup analysis according to control sources, ethnicity, and sample size, we also found lower risks of T2DM for *1/*1 genotype in Chinese (OR = 0.23, 95% CI = 0.13–0.42) and the small sample group (OR = 0.31, 95% CI = 0.11–0.89). Since *2 is considered to be an inactive allele, we compared the T2DM susceptibility between *1/*1 and *2/*2 + *1/*2. Significant decreased risks of T2DM were found in the *1/*1 genotype compared with that in the *2/*2 + *1/*2 genotype in overall analysis (OR = 0.61, 95% CI = 0.37–1.00), in control (CAD) (OR = 0.46, 95% CI = 0.23–0.93), in Chinese (OR = 0.43, 95% CI = 0.29–0.63), and in small sample groups (OR = 0.51, 95% CI = 0.33–0.77). We also compared *1/*1 with *1/*2 and *1 allele with *2 allele on the risk of T2DM. A lower risk for *1/*1 and *1 allele were found in Chinese (*1/*1 vs. *1/*2, OR = 0.48, *p* < 0.0001; *1 vs. *2, OR = 0.57, *p* < 0.00001), in normal control (*1/*1 vs. *1/*2, OR = 0.51, *p* < 0.03), and in control (CAD) (*1 vs. *2, OR = 0.49, *p* = 0.04). Interestingly, in the subgroup analysis by sample size, we found an increased risk of T2DM for the *1/*1 genotype and *1 allele in the large sample group in the heterozygous model, dominant model, and allelic model, while there was a decreased risk of T2DM for the *1/*1 genotype and *1 allele in the small sample group. No significant relationship between ALDH2 rs671 and DR was found in the dominant model (Figure 3).

### 3.3. Sensitivity Analysis

**Figure 4:**
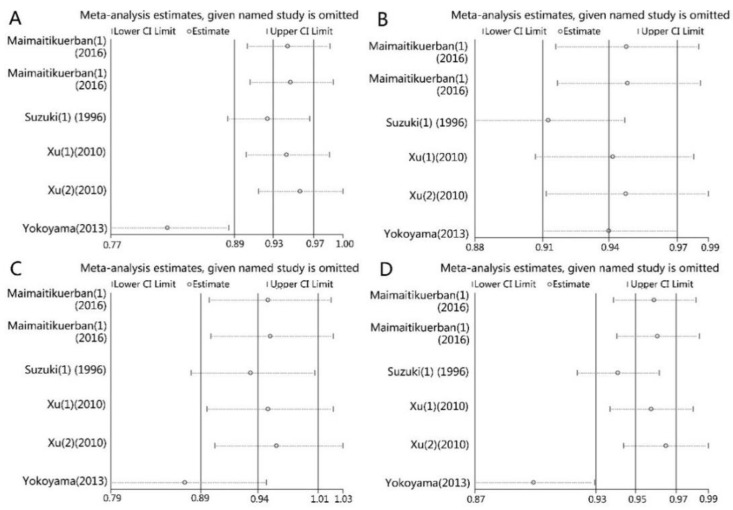
Sensitivity analysis of the influence of each study on pooled ORs and 95% CIs in different genetic models for associations of ALDH2 rs671 and DM. (**A**) *1/*1 vs. *1/*2; (**B**) *1/*1 vs. *2/*2; (**C**) *1/*1 vs. *1/*2 + *2/*2; (**D**) *1 vs. *2.

We excluded one single study at each time to evaluate the influence of each study on the pooled ORs and 95% CIs. For the relationship between ALDH2 rs671 and T2DM susceptibility, the omission of the study of Yokoyama (2013) [20] caused a significant change of pooled ORs and CIs in all genetic models except the homozygous model (Figure 4A,C,D, and Table 4). No significant change was found for the association between ALDH2 rs671 and DR susceptibility in the same analysis.

### 3.4. Publication Bias

In order to evaluate the publication bias, we conducted Egger’s test and Begg’s test. The *p*-values of both tests were shown in Table 5. Significant bias was found in Egger’s test for ALDH2 r671 and T2DM in all genotypes except the homozygous type (*p* < 0.05). We also found an obvious asymmetry in Begg’s funnel plots, indicating an obvious publication bias existing in the involved studies. No significant asymmetry was found for studies on ALDH2 rs671 and DR.

### 3.5. Source of Heterogeneity

We found a high heterogeneity between the involved studies during the meta-analysis. In order to figure out the source of the heterogeneity, subgroup analyses by source of control, ethnicity, and sample size were performed. However, as shown in Table 3, the *I*^2^ % values were greater than 50% in all subgroups, which indicated that none of them were the source of heterogeneity.

## 4. Discussion

In the present meta-analysis, we found a significantly lower risk of T2DM for ALDH2 *1/*1 genotype in the homozygous and dominant model (OR = 0.31 and OR = 0.61 respectively). Subgroup analysis by ethnicity discovered a drastic decrease in the risk for T2DM among Chinese. No significant relationship between ALDH2 rs671 and DR was found in the dominant model.

For the rs671 polymorphism of the ALDH2 gene, *1 was considered to be an active form and *2 to be an inactive form. ALDH2 was first known as a key enzyme for alcohol metabolism, especially in East Asians. Previous studies indicated that the *1/*2 or *2/*2 genotype decreased the activity of the ALDH2 enzyme. The *2/*2 lost almost all of the enzyme activity, while *1/*2 showed only 6.3% of the activity compared with the *1/*1 genotype [6]. ALDH2 is an enzyme that detoxifies reactive aldehydes, such as methylglyoxal and 4-hydroxynonenal. These aldehydes usually come from lipids and glucose. It was reported that the methylglyoxal and 4-hydroxynonenal caused the protein carbonylation and mitochondrial dysfunction, forming advanced glycation end products (AGEs) [10,11]. The lack of ALDH2 activity caused by the allele *2 increases the levels of acetaldehydes and other reactive aldehydes, and subsequently increases the amount of AGEs. Other studies also proved that the defect of ALDH2 induced oxidative stress and mitochondrial DNA mutation and deletion, which might be responsible for the formation of T2DM [23,24]. In our meta-analysis, an overall decrease in T2DM was found for ALDH2 *1/*1 genotype in homozygous and dominant models, which might be interpreted as the accumulation of aldehydes caused by the inactive *2 allele in ALDH2 enzyme.

In diabetes mellitus patients, hyperglycemia leads to both micro- and macrovascular complications, including diabetic retinopathy [2,25]. The acetaldehyde is a substance with high reactivity and mutagenesis. The accumulation of acetaldehyde could induce acute cardiovascular reactions, such as facial flushing, tachycardia, and orthostatic hypotension [20,26,27]. Rs671 mutation leads to ALDH2 dysfunction and results in the accumulation of aldehydes. Reactive aldehydes induce the formation of DR in T2DM patients through the inflammation process and cause deterioration of the vasodilator functions of the retinal vascular [28,29]. Therefore, ALDH2 allele *2 would increase the risk of complications of DM such as DR. Interestingly, the ALDH2 *2 is only related to the DR in drinkers, which suggests the reactive aldehydes derived from alcohol play key roles in DR [10]. Our analysis indicated that ALDH2 polymorphism was not pivotal for DR susceptibility. Since not all of the involved studies provided the data of drinking, we are not able to determine the effects of drinking on the relationship. Further studies and analysis are still needed to clarify this problem.

Interestingly, the *2 allele resulted in a drastically higher level of acetaldehyde in the blood and subsequently inhibited the consumption of alcohol [13]. Alcoholism was more frequently found in subjects with ALDH2 *1/*1, was significantly lower among those with ALDH2 *1/*2, and no alcoholics were found with the ALDH2 *2/*2, genotype [13]. However, studies showed that ALDH2 polymorphism was a risk factor for T2DM independent of alcohol consumption [5,12].

We found an obvious heterogeneity in the present meta-analysis for ALDH2 rs671 and DM. We have conducted subgroup analyses by source of control, ethnicity, and sample size to explore the source of this heterogeneity. However, none of them were responsible for it. Interestingly, in the study of Xu (2010) [19], the association between ALDH2 polymorphism and T2DM was only found in female patients. Moreover, the ALDH2 mutation is firmly related with ethnicity. The *2 allele of ALDH2 is very common in East Asians (about 30%–50%) compared with Caucasians (lower than 5%) [30]. The frequency of *2 allele is also different in various regions of one country. For instance, in Han Chinese people, the ALDH2 *2 frequency for people from Shanghai is about 19.7%, while it is 14.7% for people from Shandong province [31]. As shown in the research of Hui Li [32], the highest frequencies appeared in Southeast China, among the Han Chinese in Fujian and Guangdong provinces, decreasing gradually to the north and west. The allele frequencies in Han Chinese populations range from 9% to 40.9%. In our present meta-analysis, we investigated the population from Xinjiang and Shandong provinces of China, Japan, and Korea. Even though they are all Asians, they might have different genetic backgrounds. Therefore, the gender or origination of subjects might be the sources of the observed heterogeneity. However, we do not have enough data from the studies included in the present meta-analysis.

We found an obvious publication bias in Egger’s test and Begg’s test for ALDH2 rs671 and DM in the present study. After a comprehensive literature search, all the published studies were involved. However, most of the studies included in the present study had a small sample size. The small-sample effect might be responsible for the bias. We found a significant influence of the study of Yakoyama (2013) [20] on the pooled ORs in the present analysis (Table 4, Figure 4). After comparison of it with other studies, we found all the subjects included in the study were alcoholic men. That may be the cause for this difference.

The results of the present meta-analysis should be interpreted with caution as they have the following limitations: Firstly, the number of patients involved was relatively small. In the present meta-analysis, only 6 studies were included for ALDH2 polymorphism and T2DM (1247 cases and 2817 controls), and another 4 studies were included for ALDH2 polymorphism and DR susceptibility (1127 cases and 3877 controls). Secondly, a big heterogeneity and publication bias existed among the involved studies, which might affect our results. Thirdly, DM is a multi-factorial disease; the environmental factors may play important roles in its pathogenesis. In the present meta-analysis, most of the included studies lacked sufficient data on environmental exposure, such as alcohol consumption, smoking, hyperlipidemia, body mass, fasting plasma glucose, and the complications of DM. Finally, DM is a multi-gene disease which may be related with gene-gene or gene-environment interactions. However, no such information was available in the included studies.

## 5. Conclusions

In conclusion, the present meta-analysis indicated a significantly lower risk of T2DM for the *1 allele and *1/*1 genotype of ALDH2 rs671, especially among Chinese. No significant relationship between ALDH2 rs671 and DR was found in the dominant model. Thus, ALDH2 rs671 might be suggested as a predictor for the risk of T2DM. However, because of heterogeneity among the studies involved, the results of the present meta-analysis should be interpreted with caution. Further studies with large samples, including environmental factors and clinicopathological characteristics are needed to evaluate the association between ALDH2 rs671 and T2DM.

## Figures and Tables

**Table 1:** Characteristics of eligible studies included in the meta-analysis.

**ALDH2 rs671 and T2DM**							
**Author**	**Year**	**Country**	**Ethnicity**	**Genotyping Methods**	**Sex Ratio (Male %) (Case/Control)**	**Mean Age (Case/Control)**	**Quality Score**
Maimaitikuerban (1) [18]	2016	China	Asian	PCR-RFLP	96.67%/75.21%	66/60	10
Maimaitikuerban (2) [18]	2016	China	Asian	PCR-RFLP	96.67%/64.34%	66/59	10
Suzuki (1) [12]	1996	Japan	Asian	PCR-RFLP	70.6%/51.8%	not mentioned	9
Xu (1) [19]	2010	China	Asian	PCR-sequencing	70.8%/63.2%	61.7 ± 10.6/60.9 ± 10.2	12
Xu (2) [19]	2010	China	Asian	PCR-sequencing	70.8%/50.8%	61.7 ± 10.6/61.4 ± 10.0	12
Yokoyama [20]	2013	Japan	Asian	PCR-RFLP	100%/100%	57.7 ± 0.5/56.0 ± 0.2	9
**ALDH2 rs671 and DR**							
**Author**	**Year**	**Country**	**Ethnicity**	**Genotyping Methods**	**Sex Ratio (Male:Female) (Case/Control)**	**Age (Case/Control)**	**Quality Score**
Morita [13]	2013	Japan	Asian	Taqman	not mentioned	not mentioned	10
Idewaki [21]	2015	Japan	Asian	PCR-RFLP	not mentioned	not mentioned	8
Suzuki [22]	2004	Japan	Asian	PCR-RFLP	not mentioned	not mentioned	6
Suzuki (2) [12]	1996	Japan	Asian	PCR-RFLP	not mentioned	not mentioned	7

**Table 2:** Genotype frequencies of the ALDH2 rs671 polymorphism between case group and control group.

**ALDH2 rs671 and T2DM**									
**Author**	**T2DM Patients**					**Control**			**HWE of Control**
	**Total**	*1/*1	*1/*2	*2/*2	**Total**	*1/*1	*1/*2	*2/*2	
Maimaitikuerban (1) [18]	90	47	36	7	114	89	24	1	0.655
Maimaitikuerban (2) [18]	90	47	36	7	129	104	24	1	0.763
Suzuki (1) [12]	170	98	64	8	461	268	161	32	0.251
Xu (1) [19]	257	131	111	15	285	177	100	8	0.165
Xu (2) [19]	257	131	111	15	309	208	95	6	0.195
Yokoyama [20]	383	340	43	0	1519	1265	254	0	<0.0001
**ALDH2 rs671 and DR**									
**Author**	**DR Patients**				**Control**				**HWE of Control**
	**Total**	*1/*1	*1/*2 + *2/*2		**Total**	*1/*1	*1/*2 + *2/*2		
Morita [13]	52	26	26		182	117	65		N/A
Idewaki [21]	949	539	410		3451	1863	1588		N/A
Suzuki [22]	51	26	25		107	59	48		N/A
Suzuki (2) [12]	75	42	33		137	78	59		N/A

**Table 3:** Pooled odds ratios (ORs) and 95% confidence intervals (CIs) of the association between ALDH2 rs671, T2DM, and DR.

**Genetic Model**		**ALDH2 rs671 with T2DM**				
		**N**	**OR (95% CI)**	***p*-Value**	***I*^2^ (%)**	**Q Value**
*1/*1 vs. *1/*2	Overall	6	0.64 (0.41, 1.02)	0.06	87	37.16
	Control (CAD)	2	0.72 (0.38, 1.36)	0.31	90	3.04
	Normal control	4	0.51 (0.28, 0.95)	0.03	67	30.02
	Chinese	4	0.48 (0.34, 0.67)	<0.0001	53	6.41
	Japanese	2	1.21 (0.71, 2.07)	0.48	78	4.48
	Large sample	1	1.59 (1.12, 2.24)	0.009	N/A	N/A
	Small sample	5	0.55 (0.38, 0.78)	0.0009	70	13.33
*1/*1 vs. *2/*2	Overall	6	0.31 (0.11, 0.89)	0.03	74	15.57
	Control (CAD)	2	0.23 (0.05, 1.07)	0.06	51	2.03
	Normal control	4	0.35 (0.07, 1.83)	0.21	83	12.01
	Chinese	4	0.23 (0.13, 0.42)	<0.00001	22	3.86
	Japanese	2	1.46 (0.65, 3.28)	0.36	N/A	N/A
	Large sample	1	Not estimable	N/A	N/A	N/A
	Small sample	5	0.31 (0.11, 0.89)	0.03	74	15.57
*1/*1 vs. *1/*2 + *2/*2	Overall	6	0.61 (0.37, 1.00)	0.05	89	46.23
	Control (CAD)	2	0.46 (0.23, 0.93)	0.03	76	4.17
	Normal control	4	0.69 (0.35, 1.37)	0.29	92	36.64
	Chinese	4	0.43 (0.29, 0.63)	<0.0001	65	8.58
	Japanese	2	1.25 (0.78, 2.01)	0.36	73	3.65
	Large sample	1	1.59 (1.12, 2.24)	0.009	N/A	N/A
	Small sample	5	0.51 (0.33, 0.77)	0.002	80	20.12
*1 vs. *2	Overall	6	0.72 (0.50, 1.06)	0.10	87	37.33
	Control (CAD)	2	0.49 (0.25, 0.98)	0.04	81	5.39
	Normal control	4	0.87 (0.54, 1.39)	0.56	88	24.50
	Chinese	4	0.57 (0.48, 0.68)	<0.00001	46	5.59
	Japanese	2	1.26 (0.87, 1.83)	0.23	65	2.83
	Large sample	1	1.53 (1.10, 2.14)	0.01	N/A	N/A
	Small sample	5	0.63 (0.45, 0.87)	0.005	77	17.64
**Genetic Model**		**ALDH2 rs671 with DR**				
		**N**	**OR (95% CI)**	***p*-Value**	***I*^2^ (%)**	**Q Value**
*1/*1 vs. *1/*2 + *2/*2	Overall	4	1.06 (0.93, 1.22)	0.37	43	5.25
	Large sample	1	0.56 (0.30, 1.04)	0.06	N/A	N/A
	Small sample	3	1.10 (0.96, 1.26)	0.18	0	1.73

**Table 4:** The pooled ORs and 95% CIs of the association between ALDH2 rs671 and DM after removal of the work of Yakoyama (2013) [20].

Genotype Model	Number of Studies	OR (95% CI)	*p*-Value	*I*^2^ (%)
*1/*1 vs. *1/*2	5	0.55 (0.38, 0.78)	0.0009	70%
*1/*1 vs. *2/*2	5	0.31 (0.11, 0.89)	0.03	74%
*1/*1 vs. *1/*2 + *2/*2	5	0.51 (0.33, 0.77)	0.002	80%
*1 vs. *2	5	1.17 (1.01, 1.35)	0.04	47%

**Table 5:** Begg’s and Egger’s test for funnel plot asymmetries.

Group	ALDH2 rs671 and T2DM (*p*-Value)				ALDH2 rs671 and DR (*p*-Value)
	*1/*1 vs. *1/*2	*1/*1 vs. *2/*2	*1/*1 vs. *1/*2 + *2/*2	*1 vs. *2	*1/*1 vs. *1/*2 + *2/*2
Begg’s test	0.260	0.806	0.452	0.452	0.308
Egger’s test	0.005	0.406	0.008	0.015	0.145
